# Supplementary material for: Tofogliflozin Delays Portal Hypertension and Hepatic Fibrosis by Inhibiting Sinusoidal Capillarization in Cirrhotic Rats
Source: Cells. 2024 Mar 19;13(6):538. doi: 10.3390/cells13060538 (PMC10968969; doi:10.3390/cells13060538)
Supplement: Supplementary file 1 [file cells-13-00538-s001.zip › cells-2870096-supplementary.pdf]

Supplementary figures

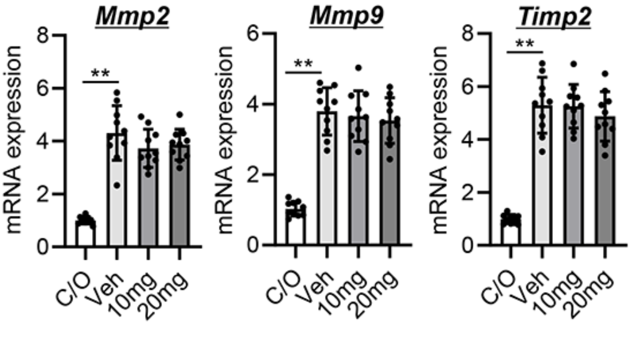

Figure S1. Effect of tofogliflozin on MMPs and Timp2 expression. Relative mRNA levels of *Mmp2*, *Mmp9*, and *Timp2* in the liver of experimental rats. *Gapdh* was used as an internal control for qRT-PCR. Quantitative values are indicated as fold changes to the values of C/O group. Data are the mean ± SD (n = 10). **P < 0.01, significant difference between groups by Student's t-test. N.S, not significant; C/O, corn oil-injected negative control group; Veh, CCl_4+vehicle-treated group; 10mg, CCl_4+tofogliflozin (10mg/kg/day)-treated group; 20mg, CCl_4+tofogliflozin (20mg/kg/day)-treated group.

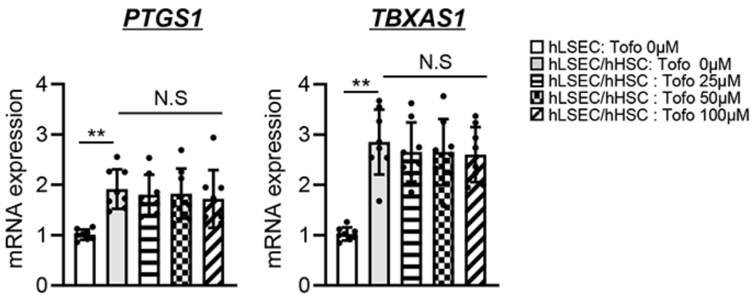

Figure S2. Effect of tofogliflozin on COX-1 and TXA2 expression in LSEC co-cultured with activated HSC. Effect of tofogliflozin (Tofo) on mRNA levels of *PTGS1* and *TBXAS1*, encoding cyclooxygenase (COX-1) and thromboxane A2 (TXA2) respectively, in activated human HSC (Ac-hHSC)-stimulated human liver sinusoidal endothelial cell (hLSEC). *GAPDH* was used as internal control for qRT-PCR. Quantitative values are indicated as fold changes to the values of mono-cultured LSEC without tofogliflozin treatment (hLSEC: Tofo 0μM) group. Data are the mean ± SD (n = 8). **P < 0.01, significant difference between groups by Student's t-test.

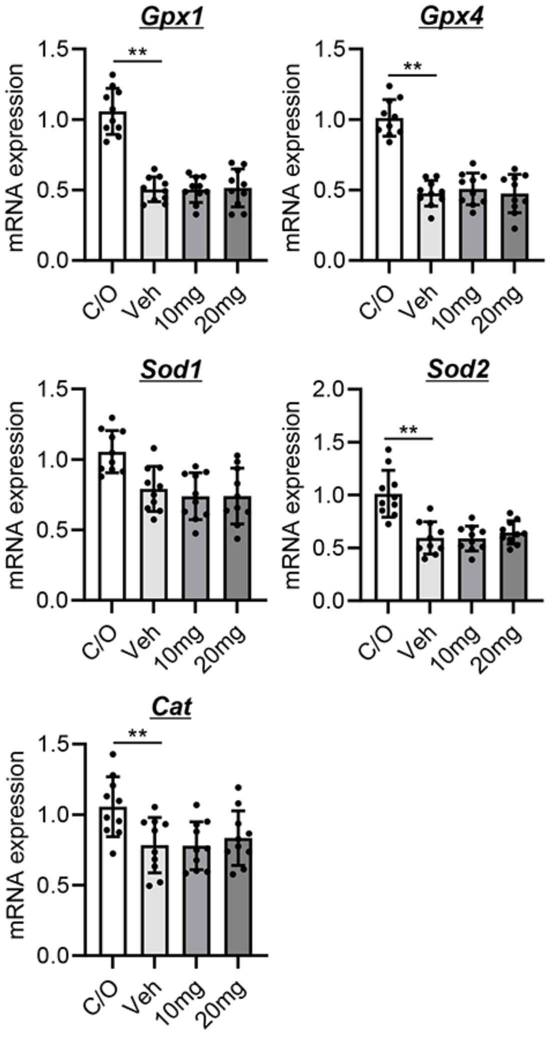

Figure S3. Effect of tofogliflozin on the anti-oxidant gene expression in the liver of CCl_4-treated rats. Relative mRNA levels of *Gpx1*, *Gpx4*, *Sod1*, *Sod2* and *Cat* in the liver of experimental rats. *Gapdh* was used as an internal control for qRT-PCR. Quantitative values are indicated as fold changes to the values of C/O group. Data are the mean ± SD (n = 10). **$P < 0.01$, significant difference between groups by Student's t-test. N.S, not significant; C/O, corn oil-injected negative control group; Veh, CCl_4+vehicle-treated group; 10mg, CCl_4+tofogliflozin (10mg/kg/day)-treated group; 20mg, CCl_4+tofogliflozin (20mg/kg/day)-treated group.

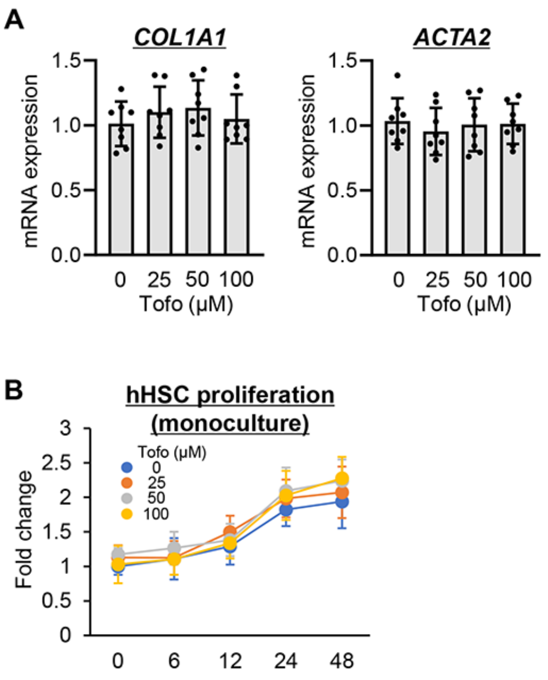

Figure S4. Direct effect of tofogliflozin on the phenotypes of activated HSC. (A) Effect of Tofo on the profibrogenic markers expression in activated human hepatic stellate cell (Ac-hHSC) under mono-culture condition. *GAPDH* was used as internal control for qRT-PCR. (B) Effect of Tofo on cell proliferation in in Ac-hHSC under mono-culture condition. Quantitative values are indicated as fold changes to the values of Tofo (0 μM) treatment group. Data are the mean ± SD (n = 8). Significant difference between groups was determined by Student's t-test.

Supplementary Table S1. List of primers used in q-PCR.

Gene	Sense (5'-3')	Antisense (5'-3')
	Rat	
Cth	CCATCCACGTGGGACAAGAG	GGATTTCCAGAGCGGCTGTA
Ddah1	CGTGGCCGATTCTTTGCATT	CATGCCCTTTGCTGGGGATA
Gch1	TCACCTGGTCCCATTTGTGG	TGGCAAGTTTGCTGAGACCA
Vegfa	TATATCTTCAAGCCGTCCTGTG	TCTCCTATGTGCTGGCTTTG
Vwf	CCTTGTGAAGTGGCTCGTCT	GCAAGTTGCAGTTGACCAGG
Ang1	CAGCTTCTCCAACAGACAAATG	ACGAGTAACCAAGCCTTGAAG
Ang2	ATCTTGTCTTGGCCTCAGCC	AATGTGTAGCTGCAGGGTCC
Adgre1	AATCGCTGCTGGCTGAATACGG	CCAGGCAAGGAGGGCAGAGTT
Tnfa	GGCTTTCGGAACTCACTGGA	CCCGTAGGGCGATTACAGTC
Il6	CACTTCACAAGTCGGAGGCT	AGCACACTAGGTTTGCCGAG
Il1b	TTGAGTCTGCACAGTTCCCC	GTCCTGGGGAAGGCATTAGG
Col1a1	GGAGAGAGCATGACCGATGG	GGTGGGAGGGAACCAGATTG
Acta2	GGCATCCACGAAACCACCTA	AGGGCTAGAAGGGTAGCACA
Pdgfrb	CAGCAAAATAACAGGACAGCG	GCAATAGCACGAACAGCAAC
Tgfbr1	ACTCCCAACTACAGAAAAGCA	CAACTTCTTCTCCCCGCCAT
Nrp1	CACCCGGTCTTCCATAAGGG	AAATCCTCAGCCGGTCTTGG
Lgals1	TCGCTTCAATCATGGCCTGT	GAAGCGGGGGTTGAAGTGTA
Mmp13	TCCATCCCGAGACCTCATGT	CTCAAAGTGAACCGCAGCAC
Timp1	TAAAGCCTGTAGCTGTGCCC	AGCGTCGAATCCTTTGAGCA
Mmp2	GGTGGCAATGGAGATGGACA	CCCGGTCATAATCCTCGGTG
Mmp9	GATCCCCAGAGCGTTACTCG	GTTGTGGAAACTCACACGCC
Timp2	CCTCTTCGCCCCTTGACAAA	CCTCTTGATGGGGTTGCCAT
Gpx1	CAGTCCACCGTGTATGCCTT	GTAAAGAGCGGGTGAGCCTT
Gpx4	ATTCCCGAGCCTTTCAACCC	TATCGGGCATGCAGATCGAC
Sod1	TAACTGAAGGCGAGCATGGG	TCCCAATCACACCACAAGCC
Sod2	ACCGAGGAGAAGTACCACGA	CCTGAACCTTGGACTCCCAC
Cat	TTTTCACCGACGAGATGGCA	AAGGTGTGTGAGCCATAGCC
Gapdh	AGCTTGTCATCAACGGGAAG	TTTGATGTTAGTGGGGTCTCG
	Human	
SGLT2	GGAGATGAATGAGCCCCAGG	GAGGCTGTGGCTTATGGTGT
CD34	CTCCAGCTGTGCGGAGTTTA	TAATAAGGGTCTTCGCCCAGC

VCAM1	CGAATGAGGGGACCACATCTA	CGCTCAGAGGGCTGTCTATC
CD32b	AGCGGATTTCAGCCAATCCC	TGGATGTGGAACGGAAGAGC
ET1	GCTGCCTTTTCTCCCCGTT	GGATGCTCCTGCTCTGATCC
GPX1	TATCGAGAATGTGGCGTCCC	TCTTGGCGTTCTCCTGATGC
GPX4	TCACCAAGTTTGGACACCGT	ATAGTGGGGCAGGTCCTTCT
SOD1	AAAGATGGTGTGGCCGATGT	CAAGCCAAACGACTTCCAGC
SOD2	GCACTAGCAGCATGTTGAGC	GCCTGTTGTTCCTTGCAGTG
CAT	AGTGATCGGGGGATTCCAGA	CCACCCTGATTGTCCTGCAT
COL1A1	TGACGAGACCAAGAACTGCC	CCATTCTTTCCAGGGGGACC
ACTA2	CACGATGTACCCTGGGATCG	GCCGATCCACACCGAGTATT
TGFB1	ACCTGCCACAGATCCCCTAT	GAGCAACACGGGTTCAGGTA
PTGS1	CGGAGTCTCTTGCTCTGGTT	GGGGGTAGTGCATCAACACA
TBXAS1	TTTGCTTGGTTGCCTGTTCC	AAAAACAGAACGCTGTCGGC
GAPDH	AATGGGCAGCCGTTAGGAAA	GCGCCCAATACGACCAAATC
